# Charge Transport through Single-Molecule Junctions with σ-Delocalized Systems

**DOI:** 10.1021/jacs.4c06732

**Published:** 2024-07-03

**Authors:** Shintaro Fujii, Saya Seko, Taichi Tanaka, Yuki Yoshihara, Shunsuke Furukawa, Tomoaki Nishino, Masaichi Saito

**Affiliations:** † Department of Chemistry, School of Science, Tokyo Institute of Technology, 2-12-1 W4-10 Ookayama, Meguro-ku, Tokyo 152-8551, Japan; ‡ Department of Chemistry, Graduate School of Science and Engineering, Saitama University, Shimo-okubo, Sakura-ku, Saitama-city, Saitama 338-8570, Japan

## Abstract

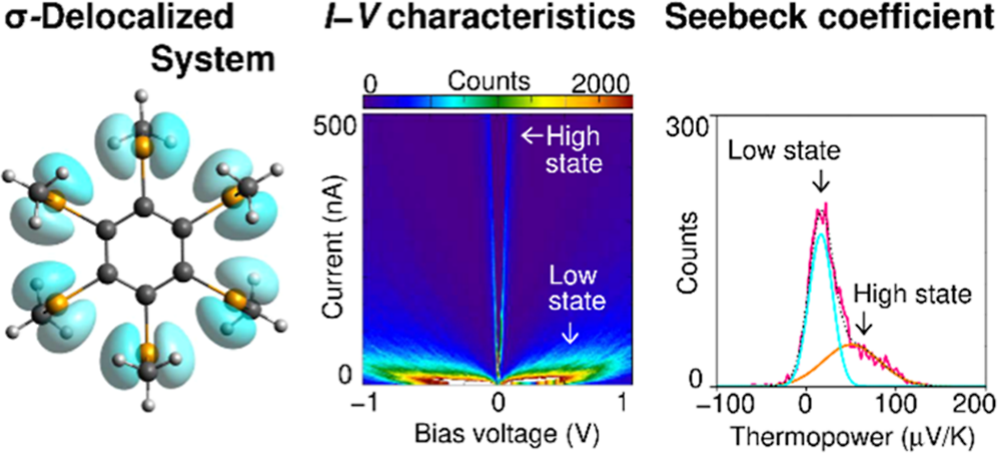

Single-molecule junctions, formed by a single molecule bridging a gap between two metal electrodes, are attracting attention as basic models of ultrasmall electronic devices. Although charge transport through π-conjugated molecules with π-delocalized system has been widely studied for a number of molecular junctions, there has been almost no research on charge transport through molecular junctions with a σ-delocalized orbital system. Compounds with hexa-selenium-substituted benzene form a σ-delocalized orbital system on the periphery of the benzene ring. In this study, we fabricated single-molecule junctions with the σ-delocalized orbital systems arising from lone-pair interactions of selenium atoms and clarified their electronic properties using the break-junction method. The single-molecule junctions with the σ-orbital systems show efficient charge transport properties and can be one of the alternatives to those with conventional π-orbital systems as minute electronic conductors.

## Introduction

**Figure 1:**
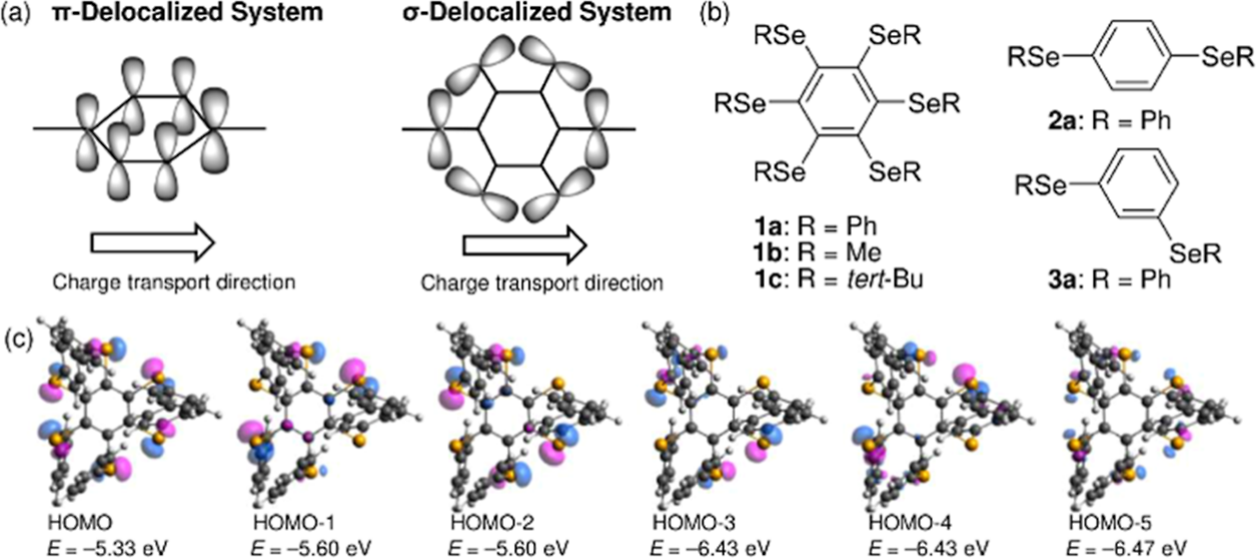
(a) Schematic illustration of charge transport through π- and σ-delocalized systems. The p-orbitals are aligned perpendicular and parallel to the charge transport direction for π-delocalized and σ-delocalized systems, respectively. (b) Chemical structures of molecules, **1a–c**, **2a**, and **3a** (Supporting Information 1) used for the present study. (c) Highest occupied molecular orbitals (HOMOs) of **1a**. Gray, white, and yellow balls correspond to C, H, and Se atoms, respectively (Supporting Information 2).

Recent technological advances have led to widespread research on charge transport properties at the level of individual molecules.^1−3^ To strategically pursue junctions with high electronic conductance, the use of electrically conductive π-conjugated molecular skeletons is essential. However, relying solely on conventional π-delocalized systems is not sufficient to provide structural diversity and expand architectural freedom in single-molecule junctions (SMJs). In the realm of organic chemistry, σ-delocalized systems consisting of σ-symmetric orbitals delocalized on nonbonded atoms^4,5^ appear as complementary entities to the π-delocalized systems. In the π-delocalized system, the charge transport direction of a SMJ is orthogonal to p-orbitals (Figure 1a, left), whereas p-orbitals in the σ-delocalized system are aligned parallel to its charge transport direction (Figure 1a, right). The σ- and π-delocalized systems offer the possibility of designing charge transport paths in SMJs and encourage the development of the research area of molecular electronics.

Although charge transport through single molecules characterized by the π-delocalized systems has long received considerable attention, molecular junctions characterized by the σ-delocalized systems have rarely been investigated. The present study aims to explore the effect of σ-delocalized systems on charge transport properties at the single-molecule scale. To that end, we investigate the single-molecule transport properties of compounds with six selenium (Se) atoms functionalized on a benzene ring^6^ (Figure 1b and Supporting Information 1) using the break junction method.^7,8^ The lone-pair electrons in the p-orbitals of the Se atoms create σ-delocalized orbitals in the periphery of the benzene,^4,5^ and the highest occupied molecular orbitals (HOMO(s)) display large amplitudes on the circular array of the Se atoms (Figure 1c). We demonstrate that HOMO(s) is responsible for charge transport in the SMJs with the σ-delocalized system and that these SMJs are highly conductive. This study has deepened our understanding of charge transport through SMJs with σ-delocalized systems and provides new insights into the design of molecular junctions based on σ-delocalized orbitals in addition to conventional π-delocalized orbitals.

## Results and Discussion

**Figure 2:**
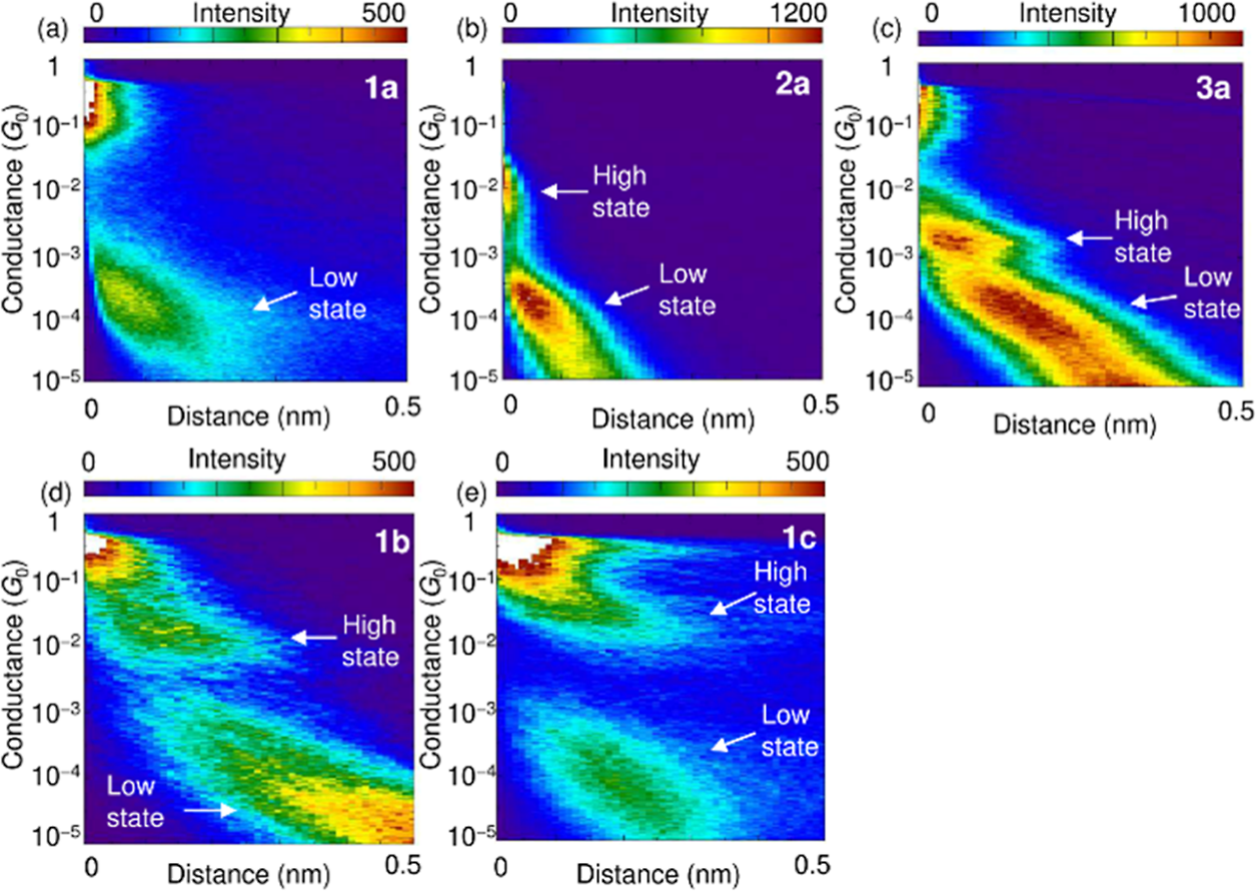
2D histograms of conductance versus stretching distance traces for single-molecule junctions of (a) **1a**, (b) **2a**, (c) **3a**, (d) **1b**, and (e) **1c** at 0.1 V, where *G*_0_ is the conductance quantum (*G*_0_ = 2*e*^2^/*h*). Linear *X*-bin-sizes of 0.005 and 0.01 nm are used for (a) and (b–e), respectively. Logarithmic *Y*-bin-size [Δlog(*G*/*G*_0_)] of 0.01 is used for (a–e). The histograms are constructed from 30,000 measurements for (a) and 10,000 measurements for (b–e). The two main distributions that represent the high and low states are indicated by arrows.

**Figure 3:**
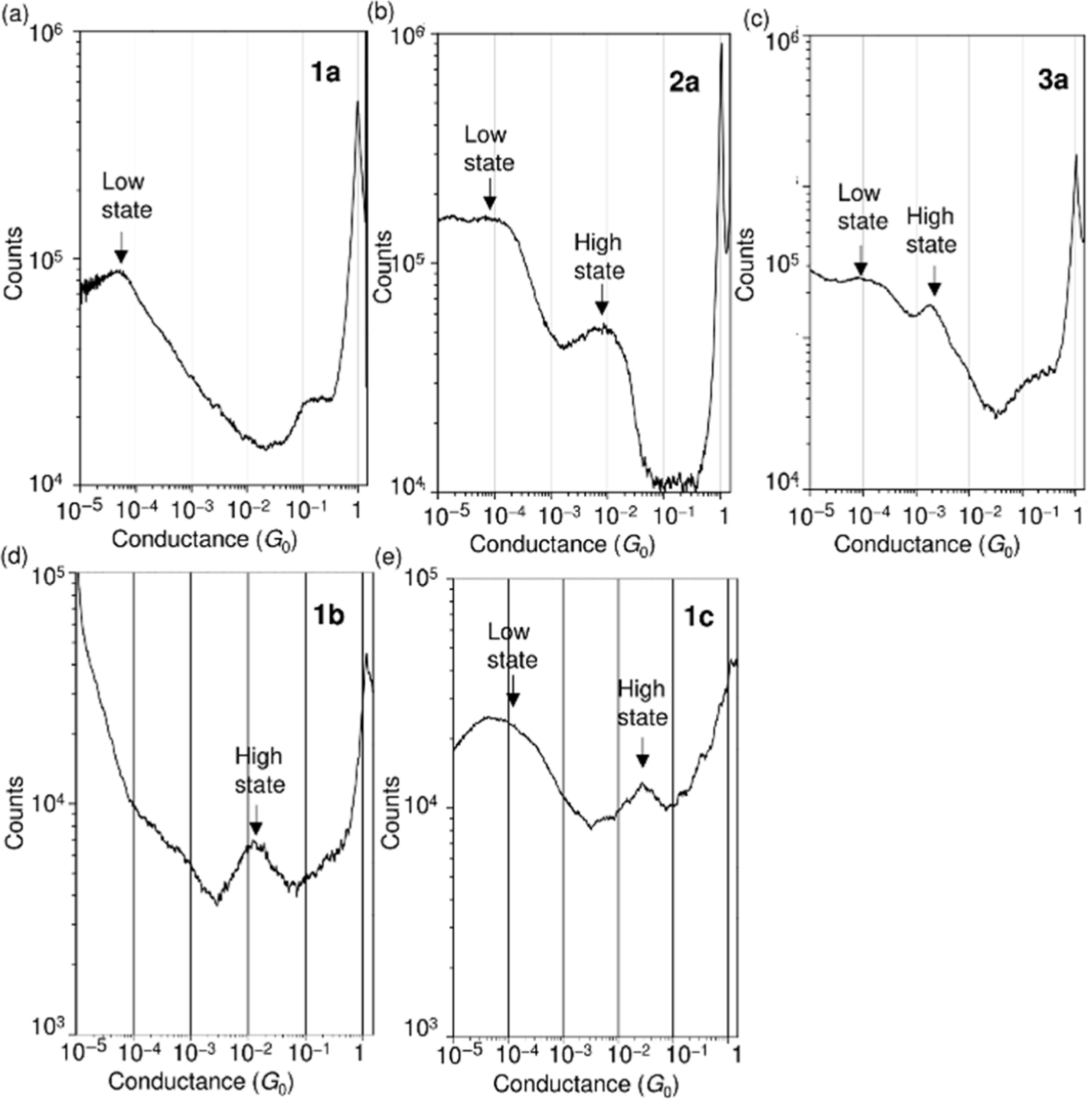
Conductance histograms of single-molecule junctions of (a) **1a**, (b) **2a**, (c) **3a**, (d) **1b**, and (e) **1c**. Logarithmic bin-size [Δlog(*G*/*G*_0_)] of 0.01 is used. The histograms were constructed from the same data set used in Figure 2. The two main distributions that represent the high and low states are indicated by arrows.

The break junction method characterizes the electronic conductance of single-molecules during the junction stretching process.^8^ Two-dimensional (2D) histograms of conductance versus stretching distance traces observed for the SMJs between two Au electrodes for **1a**, **2a**, and **3a** are characterized by one or two major distributions within the conductance range from 10^–5^ to 10^–1^*G*_0_ (Figure 2a–c and Supporting Information 2), indicating the presence of one or two types of preferential junction structures. Here, *G*_0_ is the conductance quantum (*G*_0_ = 2*e*^2^/*h*). Figure 3a–c shows conductance histograms of SMJs for **1a**, **2a**, and **3a**, respectively. The compounds **1a**, **2a**, and **3a**, respectively, have six, two, and two Se atoms that can bind to the Au electrode. To investigate the effect of the substitution position of Se atoms bound to the Au electrodes on the electronic conductance, the SMJ conductance of disubstituted benzenes (para- and meta-substituted benzenes, **2a** and **3a**) and hexasubstituted benzene (**1a**) was measured. The disubstituted benzenes exhibit high and low conductance states, with conductance differing by 1–2 orders of magnitude due to a Au–Se coordinative binding mode and a Au-Ph vdW-type binding mode, respectively (Figure 3b,c). The Se atom with lone-pair electrons can bind to an undercoordinated Au atom,^9^ while the Ph group can form van der Waals (vdW) bond to Au electrodes via direct metal–π coupling.^10,11^ In the high state, there is a marked difference in conductance depending on the substitution position of the Se atoms, with the conductance of **2a** being about four times larger than that of **3a**. The remarkable conductance difference originates from the constructive and destructive quantum interference of electron waves crossing the SMJs^12,13^ of **2a** and **3a**. In contrast to the high states, the low states of **2a** and **3a** show much lower but nearly similar conductance.

The hexasubstituted benzene (**1a**), which features the σ-delocalized system, exhibits only a low state (Figure 3a) and its conductance is similar to those of the low states for **2a** and **3a** (Figure 3b,c). The similarity of the low-state conductances of **1a** to those of **2a** and **3a** suggests that **1a**, like **2a** and **3a**, forms SMJs in a Au-Ph vdW-type binding mode. Given that **1a** has six Ph groups, it may exhibit various low-state conductances depending on the binding position of the Ph groups to the electrodes. It is possible that these low states, arising from the different binding positions, exhibit conductances below 10^–5^*G*_0_, which were not detected in the current experiment.

The six bulky Ph groups on the periphery of the benzene ring can hamper the binding of the Se atoms in the σ-delocalized system to the Au electrodes and prevent the formation of a high state with an Au–Se binding mode. To assess this steric hindrance effect, the large Ph groups on the periphery of the benzene ring in **1a** were replaced by small Me groups in **1b**. Interestingly, a high state of **1b**, which features the σ-delocalized system, shows the highest conductance (Figures 2d and 3d) compared to those of **2a** and **3a** that do not have σ-delocalized systems. The highest conductance of **1b** is due to the σ-delocalized system with the Au–Se bonding mode.^9^ Referring to the results of para-substituted **2a** and meta-substituted **3a** (Figure 3b,c), **1b** should show multiple high states, depending on the two Au–Se connection sites, such as para- and meta-connection sites, that contributed to the SMJ formation out of six possible Se connection sites. Even though the **1b** contains six Se atoms and can be connected to two Au electrodes at various connection sites, the high state exhibits a single conductance (Figure 3d). This result suggests that conductance through the molecule with the σ-delocalized system of **1b** is almost independent of the Se connection positions to the Au electrodes. A low state of **1b** is not apparent, presumably because the low state is likely below the detection limit of the present measurement system (see below). If the benzene ring of **1b** lies on the Au electrodes and forms a direct Au–π coupling, the charge transport direction is perpendicular to the benzene plane. For example, mesitylene (i.e., benzene with three methyl groups) has been reported to lie on Au electrodes and form a direct Au–π bonding mode with Au electrodes, leading to high electronic conductance above 0.1*G*_0_.^14^ Since the high state of **1b** did not show such a high electronic conductance, we can rule out the possibility that the high state of **1b** is due to direct Au–π coupling. The Se–R functional groups in **1b** are bulkier than the methyl group in mesitylene, which can prevent the benzene ring of **1b** from being parallel to the Au electrode.

**Figure 4:**
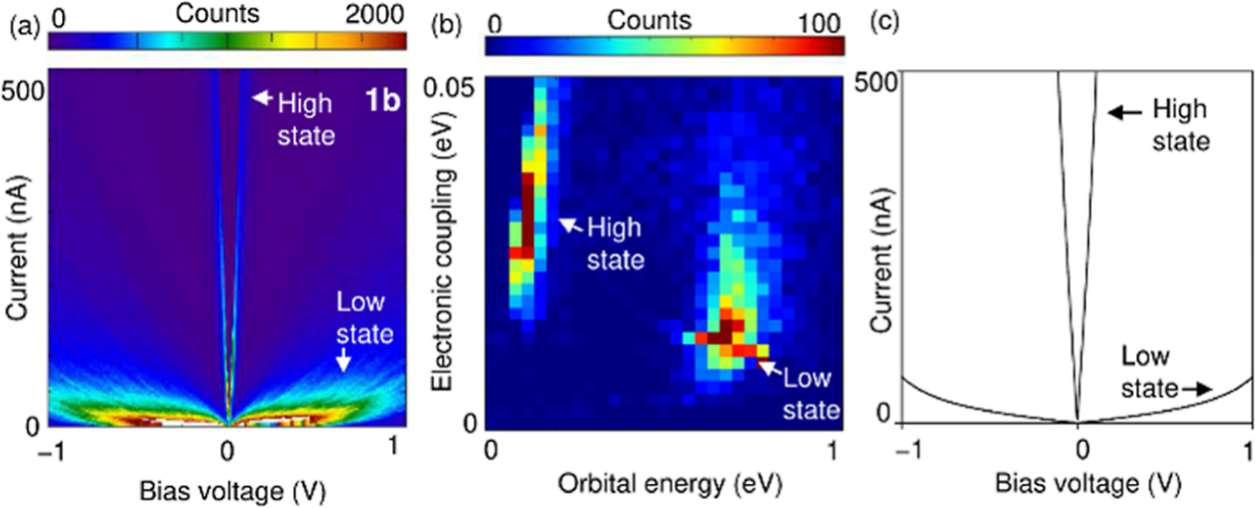
(a) 2D map of the *I*–*V* curves of single-molecule junctions of **1b**, which was constructed from 10,000 experimentally obtained *I*–*V* curves. The sizes of the *X*- and *Y*-bins are 6 mV and 1 nA, respectively. (b) 2D map of ε versus Γ for single-molecule junctions of **1**. The sizes of the *X* and *Y*-bins are 33 and 1.7 meV, respectively. (c) Theoretical *I*–*V* curves calculated from eq 1 using the parameters obtained by the curve fitting [i.e., (Γ, ε) = (0.03, 0.13 eV) and (0.01, 0.67 eV)].

To further characterize the conductance states and charge transport properties of the σ-delocalized system, current–voltage (*I*–*V*) and thermoelectric measurements of **1b** were performed. Figure 4a shows a 2D histogram constructed from 10,000 of *I*–*V* curves **1b**, featuring the high and low states. To evaluate the detailed electronic structures of the high and low states, we analyzed the *I*–*V* curves using eq 1, which represents a current passing through a SMJ, where Γ and ε are the electronic coupling across the metal–molecule interface and the energy level of a molecular orbital relative to the Fermi level of a metal electrode, respectively.^15^
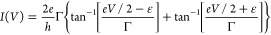
1

As shown in Figure 4b, the 2D map of Γ versus ε obtained by fitting each *I*–*V* curve using eq 1, demonstrated the two different distributions clearly and thus allowed us to determine the statistically probable values of (Γ, ε) to be (0.03, 0.13 eV) and (0.01, 0.67 eV) for the high and low states, respectively (Supporting Information 2). Figure 4c shows the theoretical *I*–*V* curves calculated from eq 1 using the fitting results. The good agreement between the theoretical and experimental *I*–*V* curves indicates the validity of the analysis in the *I*–*V* curves. The low state was not clearly observed in the low bias measurement at 0.1 V (Figure 3); however, the *I*–*V* measurement in the high bias range of ±1.0 V can resolve both high and low states of **1b** (Figure 4). Similar to **1a**, the low state of **1b** is attributed to SMJ formation with the van der Waals bonding mode via direct metal–σ coupling between the Au electrode and the Me group in **1b**. It has been reported that saturated hydrocarbons form vdW bonds with Au to form SMJs.^16^ The high state with the Au–Se coordinative binding mode is characterized by relatively large Γ and significantly small ε compared to the low state with the metal–σ binding mode. As for the electronic coupling, the Au–N coordinative binding mode is reported to have Γ = 0.035 eV,^15^ and the Au–Se coordinative binding mode in the high state of **1b** shows a similar value (Γ = 0.03 eV). For the metal–σ binding mode in the low state, no reports on the electronic coupling for analogous binding modes have appeared. As discussed above, the low state of the hexasubstituted benzene may exhibit various conductances depending on the binding position of the alkyl groups to the electrodes, which remain unresolved in the *I*–*V* measurement. The wide distribution in the 2D *I*–*V* histogram at the low state (Figure 4a) may reflect differences in conductance due to differences in the binding position of and conformation of alkyl groups on Se atoms.

**Figure 5:**
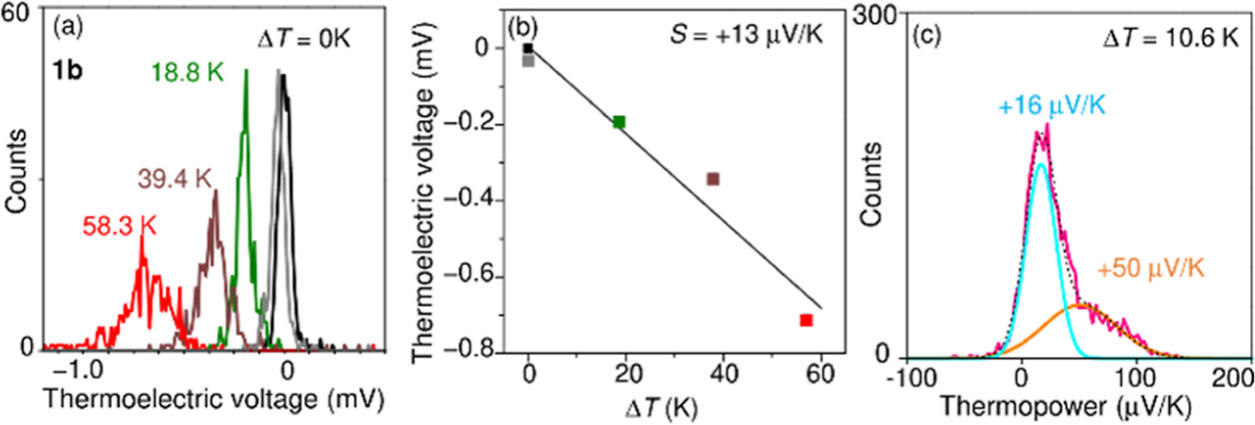
(a) Distribution of thermoelectric voltage (*V*_th_) for **1b**, measured at different temperature differences (Δ*T*). Each distribution consists of 300 measurements. (b) Plot of the peak value of *V*_th_-distribution versus Δ*T*. The peak values of *V*_th_ were obtained by fitting the distribution with the Gaussian function. (c) Distribution of thermopower (*S*) for **1b** at Δ*T* = 10.6 K. The distribution consists of 5000 measurements. The *S* value was calculated as *S* = −*V*_th_/Δ*T*. The distribution of *S* (red line) is fitted with two Gaussian functions. The dotted line is the total fitting result, and each Gaussian function is represented by blue and orange lines. The peak values are +16 and +50 μV K^–1^.

Next, to experimentally confirm that the molecular orbital that conducts charges is HOMO (Figure 1c), thermopower (*S*) was determined by applying the temperature difference (Δ*T*) across the SMJ of **1b** and measuring the thermoelectric voltage (*V*_th_).^17,18^Figure 5a shows the distribution of the measured thermoelectric voltage for **1b** at different Δ*T* across the junctions. Each distribution shows a broad peak, and this peak shifts with Δ*T*. The *V*_th_ value changes linearly with Δ*T* (Figure 5b) according to the following equation: *V*_th_ = −*S*Δ*T*. The thermopower of the SMJ was determined as +13 μV K^–1^ for **1b**, indicating that the major carrier is a hole and the conduction orbital is HOMO(s). To evaluate the thermoelectric properties of **1b** in detail, *V*_th_-measurements were repeated at a constant Δ*T*. Figure 5c shows the distribution of *S* values at Δ*T* = 10.6 K, which consists of 5000 measurements for **1b**. Because the distribution has a broad shoulder on the right side, it was fitted with two Gaussian functions. A closer examination of the statistical distribution of *S* reveals the presence of an extensive minor distribution that was not clearly visible in Figure 5a. Gaussian fitting of the major and minor distributions yields *S* of +16 and +50 μV K^–1^ corresponding to the low and high states, respectively.

**Figure 6:**
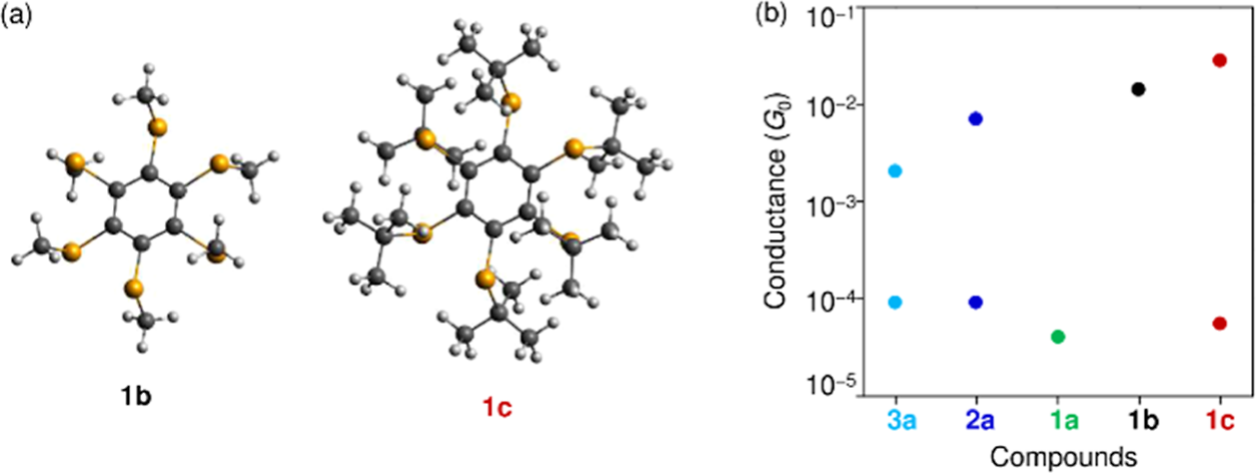
(a) Optimized geometry of **1b** and **1c** (Supporting Information 2). (b) Plot of the peak conductance for **1a**–**c**, **2a**, and **3a**. The conductance is determined as a Gaussian peak(s) in the histograms in Figure 3.

In general, the electronic conductance of a SMJ is higher as the frontier molecular orbital energy is closer to the Fermi level of metal electrodes.^15^ Substitutional groups of a molecule in a SMJ affect the energy levels of molecular orbitals, which in turn affect the single-molecule conductance. The effect of substituents has been confirmed for π-delocalized systems.^19,20^ To clarify the effect of the substituents on the charge transport properties of the σ-delocalized system (**1**), we measured the molecular conductance of **1c** where substitutional groups were changed from Me to ^*t*^Bu groups (Figures 2e and 3e). In a manner similar to **1b** (Figure 4), **1c** exhibits high and low states. Figure 6b summarizes the electronic conductance of **1a**–**c**, **2a**, and **3a**, with **1c** having the highest conductance among the high states. Since the ^*t*^Bu group is more electron-donating than the Me group, the HOMO energy level of the occupied orbitals in **1c** (−4.5 eV) is higher than that in **1b** (−5.0 eV), which is supported by theoretical calculations (Supporting Information 2). As a result, the energy level of HOMO(s) is closer to the Fermi level of the Au electrodes (−5.5 to −5.3 eV)^21,22^ and **1c** has higher electronic conductance. Interestingly, **1a** with large Ph groups cannot form a high state with an Au–Se binding mode, while **1c** with bulky ^*t*^Bu groups can form a high state. One of the possible scenarios for the preferential appearance of the high state is that deprotection of the ^*t*^Bu groups on the metal electrode surface^23^ reduces the steric hindrance effect, leading to the preferential formation of Au–Se bonding mode in the SMJ of **1c**. The elimination of ^*t*^Bu cations with three electron-donating Me groups is thermodynamically feasible on the Au electrode surface.

## Conclusions

We have investigated for the first time the charge transport properties of SMJs with the σ-delocalized systems of the six Se atoms circularly arranged on a benzene platform by the break junction method. The electronic and thermoelectric measurements indicate that SMJs with the σ-delocalized systems exhibit efficient charge transport properties with a conductance of ca. 10^–2^*G*_0_ and a relatively large thermopower of ∼50 μV K^–1^ compared to those of molecules bearing conventional π-delocalized systems in single-molecule transport studies (where |*S*| values are reported to be ∼33 μV K^–1^).^17,24^ In π-delocalized systems, the charge transport direction is orthogonal to p-orbitals, whereas p-orbitals in the σ-delocalized system are aligned parallel to their charge transport direction. π-delocalized systems such as benzene must be connected to Au electrodes via binding groups such as Se atoms, and its conductance is dependent on the binding positions to the electrodes due to the quantum interference effect. In sharp contrast, the σ-delocalized system (i.e., the circular array of the six Se atoms) binds directly to the electrodes without introducing any binding groups, and its conductance is likely to be virtually independent of the binding positions to the electrodes. This study should promote the understanding of charge transport through SMJs with σ-delocalized systems, which has been little studied to date, and provide new insights and impacts into the design of single-molecule conductors using σ-delocalized orbitals.

## Supporting Information Available

Details of synthesis, break junction measurements, and data analysis (PDF)
