# Supplementary material for: Charge Transport through Single-Molecule Junctions with σ-Delocalized Systems
Source: J Am Chem Soc. 2024 Jul 3;146(28):19566–71. doi: 10.1021/jacs.4c06732 (PMC11258778; doi:10.1021/jacs.4c06732)
Supplement: Supplementary file 1 — ja4c06732_si_001.pdf [file ja4c06732_si_001.pdf]

Supporting Information

Charge Transport through Single-Molecule Junctions with σ-Delocalized Systems

Shintaro Fujii,*† Saya Seko,‡ Taichi Tanaka,‡ Yuki Yoshihara,† Shunsuke Furukawa,‡ Tomoaki Nishino,† and Masaichi Saito,* ‡

†Department of Chemistry, School of Science, Tokyo Institute of Technology, 2-12-1 W4-10 Ookayama, Meguro-ku, Tokyo 152-8551, Japan

‡Department of Chemistry, Graduate School of Science and Engineering, Saitama University, Shimo-okubo, Sakura-ku, Saitama-city, Saitama, 338-8570, Japan

Table of contents

1. Synthesis
2. Experimental detail
   Supporting Figures
   Supporting References
3. NMR spectra of compound **3a**

1. Synthesis

Compounds **1a**,[S1] **1b**[S2] and **1c**[S3] were prepared according to the procedures in the references. Compounds **2a** and **3a** were prepared by a method similar to that for compound **1a**, shown below.

A mixture of diphenyldiselenide (1 equiv.) and sodium hydride (2 equiv.) in DMI was heated at 60 °C for 3 h, and then the mixture was cooled to room temperature. To the resulting solution of sodium phenylselenolate was added 1,4-dibromobenzene and 1,3-dibromobenzene for compounds **2a** and **3a**, respectively, and then the mixture was heated at 100 °C for 12 h. After the mixture was poured into water, and then the organic phase was extracted with chloroform. After the organic phase was dried over anhydrous magnesium sulfate, the volatile substances were removed to afford an oily residue, which was purified by silica gel column chromatography (eluent; dichloromethane : hexane = 1 : 4) to provide compounds **2a** and **3a**. The ^{1}H and ^{13}C NMR spectra of compound **2a** are identical to those previously reported.[S4] On the other hand, the ^{1}H and ^{13}C NMR spectra of compound **3a** are different from those previously reported,[S4] and we independently completely identified the product. The coupling pattern of the ^{1}H NMR signal at 7.51 ppm assignable to the C2 aromatic proton is also found in that in the ^{1}H NMR spectrum of 1,3-dibromobenzene, commercially available, indicating that compound **3a** surely possesses its 1,3-disubstituted structure. For a reference, we prepared 1,2-bis(phenylselenyl)benzene independently by our method, and the ^{1}H and ^{13}C NMR spectra are identical to those previously reported.[S5]

Compound **3a**: An orange oil, ^{1}H NMR ($CDCl_3$, 400 MHz) δ 7.14 (t, *J*=7 Hz, 1H), 7.24−7.31 (m, 6H), 7.45−7.47 (m, 4H), 7.51 (t, *J*=2 Hz, 1H); ^{13}C NMR ($CDCl_3$, 101 MHz) δ 127.75 (CH), 127.76 (CH), 129.49 (CH), 130.01 (CH), 130.36 (C), 131.05 (CH), 132.89 (CH), 133.60 (CH), 135.76 (C, *J*(C−Se) = 12 Hz); ^{77}Se NMR ($CDCl_3$, 400 MHz) δ 422.7. Anal. Calcd for $C_{18}H_{14}Se_2$: C, 55.68; H, 3.63. Found: C, 55.70; H, 3.59.

2. Experimental details

Experimental Details of Break Junction Measurements. The Au tip was prepared by mechanically cutting a Au wire (Nilaco, diameter ≈0.3 mm, purity >99.9 %) The Au(111)/mica substrates were prepared by thermal evaporation of Au on mica; Au(111) substrates were flame annealed and cleaned before use. For sample preparation, Au substrates were immersed in 0.5~1 mM toluene solution of each molecule for at least 20 minutes. After immersion, the substrates were thoroughly rinsed with toluene and dried under an inert gas flow. The electronic

measurements were performed by a commercially available ambient scanning tunnelling microscopy (STM) (MS-10 and Nanoscope V, Bruker) with a signal access module III (Bruker), an external piezo driver (M-2141, MES-TEK), and a data-acquisition-device with LabVIEW2016 (NI PXI-4461, National Instruments).

In the break junction (BJ) method,[S6] an Au-tip was repeatedly moved in and out of contact with the Au (111) substrate in the presence of the molecules. After the breakage of the Au point contact, a small gap with nano-sized electrodes was prepared between the Au tip and the Au substrate. A surface-deposited molecule can be trapped into the nanogap and a single-molecule junction (SMJ) can be fabricated using the BJ technique. Figure 2 shows 2D conductance versus stretching distance histograms of the conductance traces of the SMJs, in which electronic currents of the SMJs are repeatedly measured during the stretching process of the junctions by applying a fixed bias voltage between the Au sample electrodes and the Au-STM tip. In the 2D histograms, the stretching distance where the conductance drops blew 0.5 G_0 ($G_0 = 2e^2/h$) was set to zero, and the conductance traces overlapped each other. Therefore, the statistical distribution in the 2D histogram indicates the probable length and conductance of the SMJs. The 1D conductance histograms in Figure 3 were constructed from the same data set used in Figure 2.

In the thermoelectric measurements, the temperature of the STM tip was maintained at room temperature while that of the substrate was controlled by a Peltier device (CP0.8-31-06L, Laird Technologies) using a temperature controller (Model 331, Lake Shore Cryotronics). The substrate temperature was monitored with a resistive thermometer. Meanwhile, SMJs were made by the BJ method at a constant bias voltage of 0.5 mV. After forming the SMJ, the current-voltage (I−V) curve of the junction was measured by varying the tip bias voltage from 0.5 mV to −1.5 mV at a constant electrode spacing, while applying a constant temperature difference to the junction. The thermoelectric voltage was determined as the bias voltage shift at zero current in the I−V curve with and without a temperature gradient in the molecular junction (Figure 5). Details of the experimental setup are described in previous reports.[S7,S8]

Analysis of *I*–*V* Characteristics. I–V characteristics of the SMJs in Figure 4a were analyzed according to the Landauer-Büttiker formalism.[S9–S11] The current through single-channel SMJs can be represented by

$$I(V) = \frac{2e}{h}\int_{-\infty}^{+\infty} dE \cdot \tau(E)\left[f\left(E-\frac{eV}{2}\right)-f\left(E+\frac{eV}{2}\right)\right], \quad \text{equation (S1)}$$

$$\tau(E) = \frac{\Gamma^2}{\Gamma^2 + (E-\varepsilon)^2}, \quad \text{equation (S2)}$$

where τ and ε are the transmission probability and the energy level of the molecular orbital, respectively, and Γ is the electronic coupling across the metal-molecule interface.[S11] Here, we set the Fermi level, E_F, to zero. Because tunneling behavior is reasonably insensitive to temperature, it is convenient to work at the limit of zero temperature, where the Fermi functions become step functions. Then, the current through the SMJ is written by

$$I(V) = \frac{2e}{h}\Gamma\left\{\tan^{-1}\left[\frac{eV/2-\varepsilon}{\Gamma}\right] + \tan^{-1}\left[\frac{eV/2+\varepsilon}{\Gamma}\right]\right\}. \quad \text{equation (S3)}$$

It should be noted that the temperature effect of the Fermi-Dirac distribution is several percentage points of $I(V)$ at 300 K.[S12] 2D map of Γ versus ε in Figure 4b was obtained by fitting each I–V curve using equation (S3).

Theoretical calculations. The optimized geometries of **1a, 1b**, and **1c** were obtained by DFT calculations at the B3LYP/6-31+g(d) for C and H atoms and the B3LYP/SDD level for Se atoms using the Gaussian 16 program (Figure S1).[S13] In accordance with the literature,[S1,S14] initial geometry of each molecule was produced before the optimization.

Supporting Figures

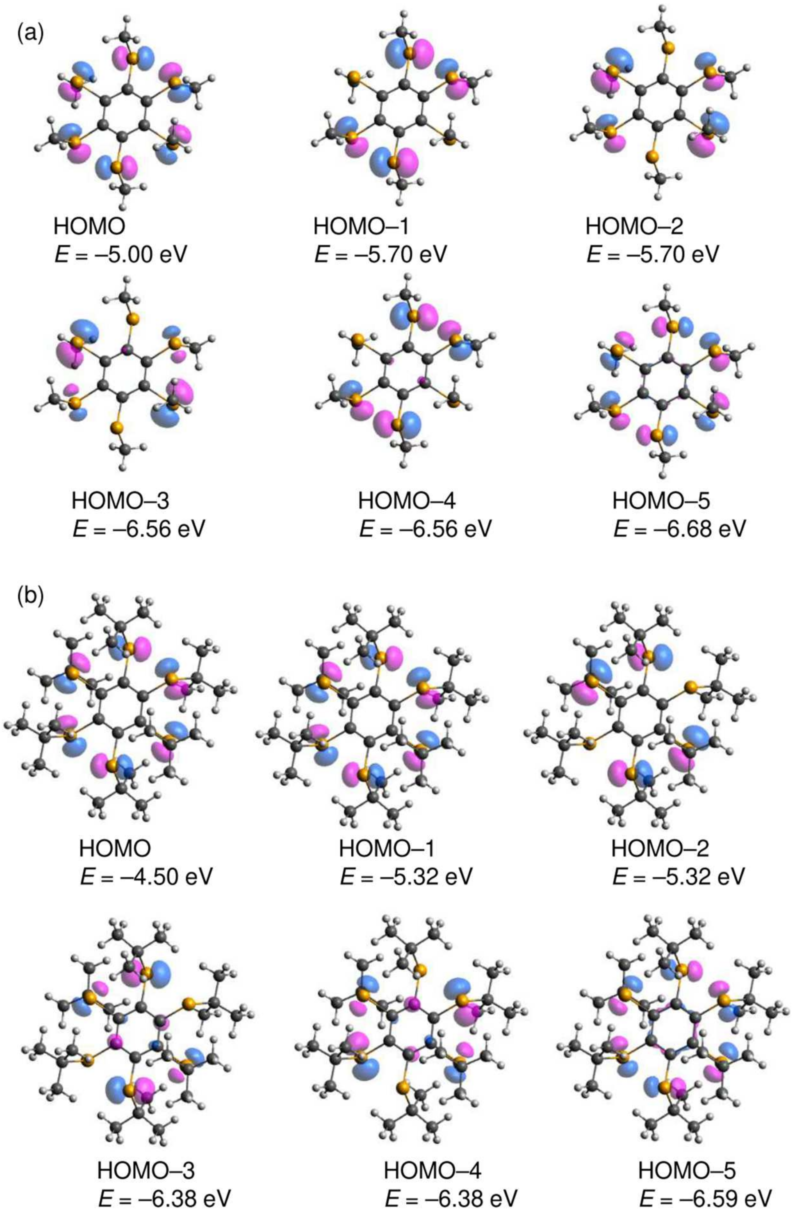

Figure S1. Optimized geometries and HOMO levels of **1b** and **1c**.

Supporting References

[S1] Furukawa, S.; Fujita, M.; Kanatomi, Y.; Minoura, M.; Hatanaka, M.; Morokuma, K.; Ishimura, K.; Saito, M. *Commun. Chem.* **2018**, *1*, 60.

[S2] Cui, Y.; Yan, J.; Chen, Z.; Zhang, J.; Zou, Y.; Sun, Y.; Xu, W. ;Zhu, D. *Adv. Sci.* **2019**, *6*, 1802235.

[S3] Turner, D. L.; Vaid, T. P. *J. Org. Chem.* **2012**, *77*, 9397–9400.

[S4] Pradhan, A. K.; Behera, P. K.; Choudhury, P.; Behera, P.; Swain, A.; Bag, P.; Pany, D.; Rout, L. *ChemistrySelect* **2023**, *8*, e202300378.

[S5] Nakanishi, W.; Hayashi, S. *J. Organomet. Chem.* **2000**, *611*, 178−189.

[S6] Xu, B.; Tao, N. J. Measurement of Single-Molecule Resistance by Repeated Formation of Molecular Junctions. *Science* **2003**, *301*, 1221−1223.

[S7] Komoto, Y.; Isshiki, Y.; S. Fujii, Nishino, T.; Kiguchi M. Evaluation of Electronic Structure of the Single-molecule Junction Based on Current-Voltage Measurement and Thermopower Measurement-Application to C_{60} Single-Molecule Junction. *Chem. Asian J.* **2017**, *12*, 440–445.

[S8] Fujii, S.; Montes, E.; Cho, H.; Yue, Y.; Koike, M.; Nishino, T.; Vázquez, H.; Kiguchi, M. Mechanically Tuned Thermopower of Single-Molecule Junctions. *Adv. Electron. Mater.* **2022**, *8*, 2200700.

[S9] Landauer, R. Spatial Variation of Currents and Fields Due to Localized Scatterers in Metallic Conduction. *IBM J. Res. Dev.* **1957**, *1*, 223−231.

[S10] Nitzan, A.; Ratner, M. A. Electron Transport in Molecular Wire Junctions, *Science* **2003**, *300*, 1384−1389.

[S11] Cuevas, J. C.; Scheer, E. Molecular Electronics, World Scientific Publishing Co. Pte. Ltd 2010.

[S12] Komoto, Y.; Fujii, S.; Nakamura, H.; Tada, T.; Nishino, T.; Kiguchi, M. Resolving Metal-Molecule Interfaces at Single-Molecule Junctions. *Sci. Rep.* **2016**, *6*, 26606.

[S13] Gaussian 16, Revision B.01, Frisch, M. J.; Trucks, G. W.; Schlegel, G. E.; Scuseria, H. B.; Robb, M. A.; Cheeseman, J. R.; Scalmani, G.; Barone, V.; Petersson, G. A.; Nakatsuji, H., Li, X.; Caricato, M.; Marenich, A.; Bloino, J.; Janesko, B. G.; Gomperts, R.; Mennucci, B.; Hratchian, H. P.; Ortiz, J. V.; Izmaylov, A. F.; Sonnenberg, J. L.; Williams-Young, D.; Ding, F.; Lipparini, F.; Egidi, F.; Goings, J.; Peng, B.; Petrone, A.; Henderson, T.; Ranasinghe, D.; Zakrzewski, V. G.; Gao, J.; Rega, N.; Zheng, G.; Liang, W.; Hada, M.; Ehara, M.; Toyota, K.; Fukuda, R.; Hasegawa, J.; Ishida, M.; Nakajima, T.; Honda, Y.; Kitao, O.; Nakai, H.; Vreven, T.; Throssell, K.; Montgomery, J. A.; Peralta, Jr., J. E.; Ogliaro, F.; Bearpark, M.; Heyd, J. J.;

Brothers, E.; Kudin, K. N.; Staroverov, V. N.; Keith, T.; Kobayashi, R.; Normand, J.; Raghavachari, K.; Rendell, A.; Burant, J. C.; Iyengar, S. S.; Tomasi, J.; Cossi, M.; Millam, J. M.; Klene, M.; Adamo, C.; Cammi, R.; Ochterski, J. W.; Martin, R. L.; Morokuma, K.; Farkas, O.; Foresman, J. B.; Fox, D. J. Gaussian, Inc., Wallingford CT, 2016.

[S14] Turner, D. L.; Vaid, T. P. Synthesis of Protected Benzenepolyselenols. *J. Org. Chem.* **2012**, *77*, 9397−9400.

1H NMR of Compound 3a

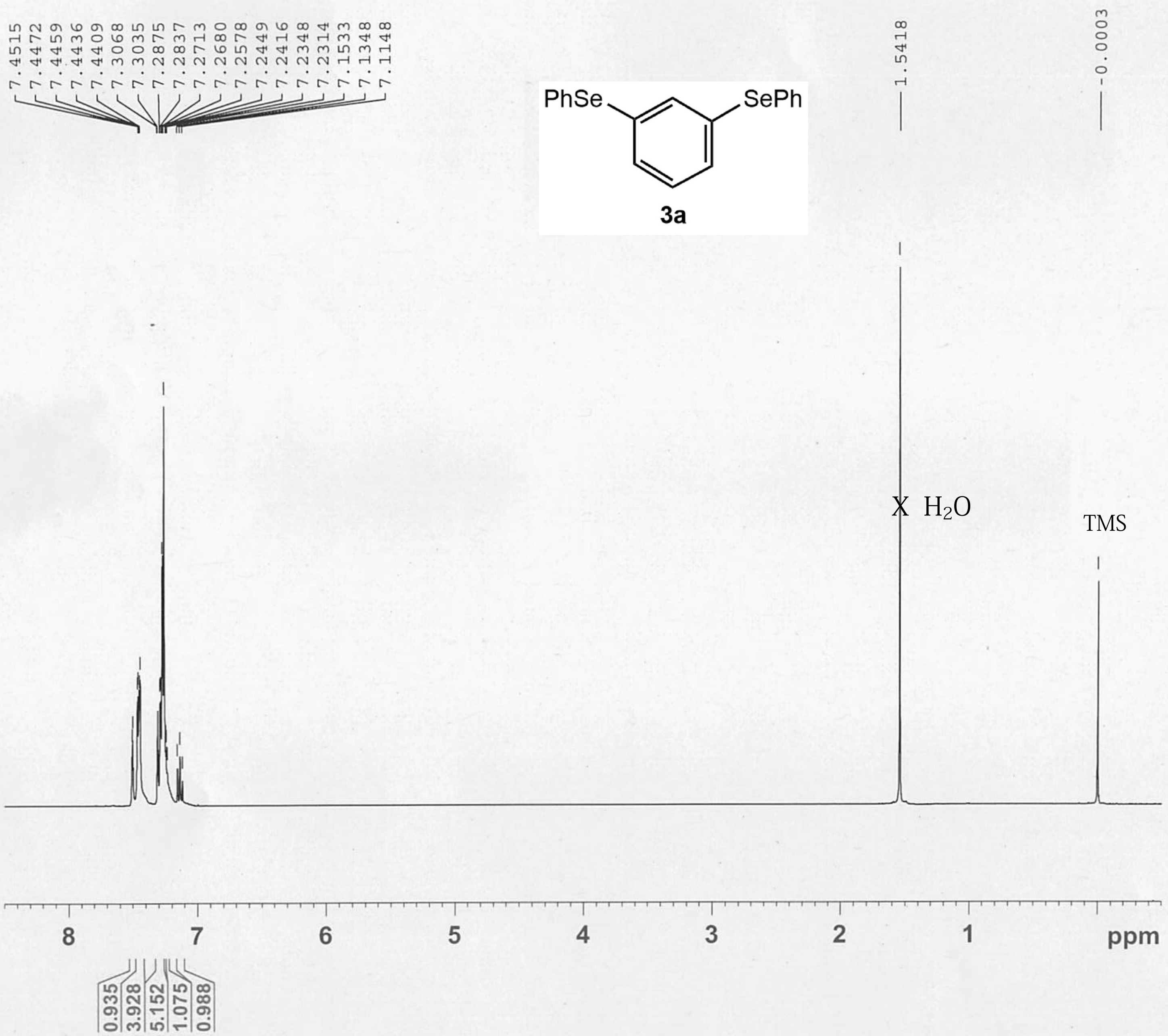


```
EXPNO              24061401
PROCNO                    1
Date_              20240614
Time                  12.33
INSTRUM               spect
PROBHD     5 mm CPQNP 1H/
PULPROG                zg30
TD                    65536
SOLVENT               CDCl3
NS                        4
DS                        2
SWH              8305.647 Hz
FIDRES           0.126734 Hz
AQ              3.9453173 sec
RG                    43.01
DW                   60.200 usec
DE                    18.00 usec
TE                    300.0 K
D1               1.00000000 sec
TD0                       1

======== CHANNEL f1 ========
SFO1          400.1324708 MHz
NUC1                     1H
P1                    15.00 usec
SI                    32768
SF            400.1300104 MHz
WDW                      EM
SSB                       0
LB                     0.30 Hz
GB                        0
PC                     1.00
```

13C NMR of Compound 3a

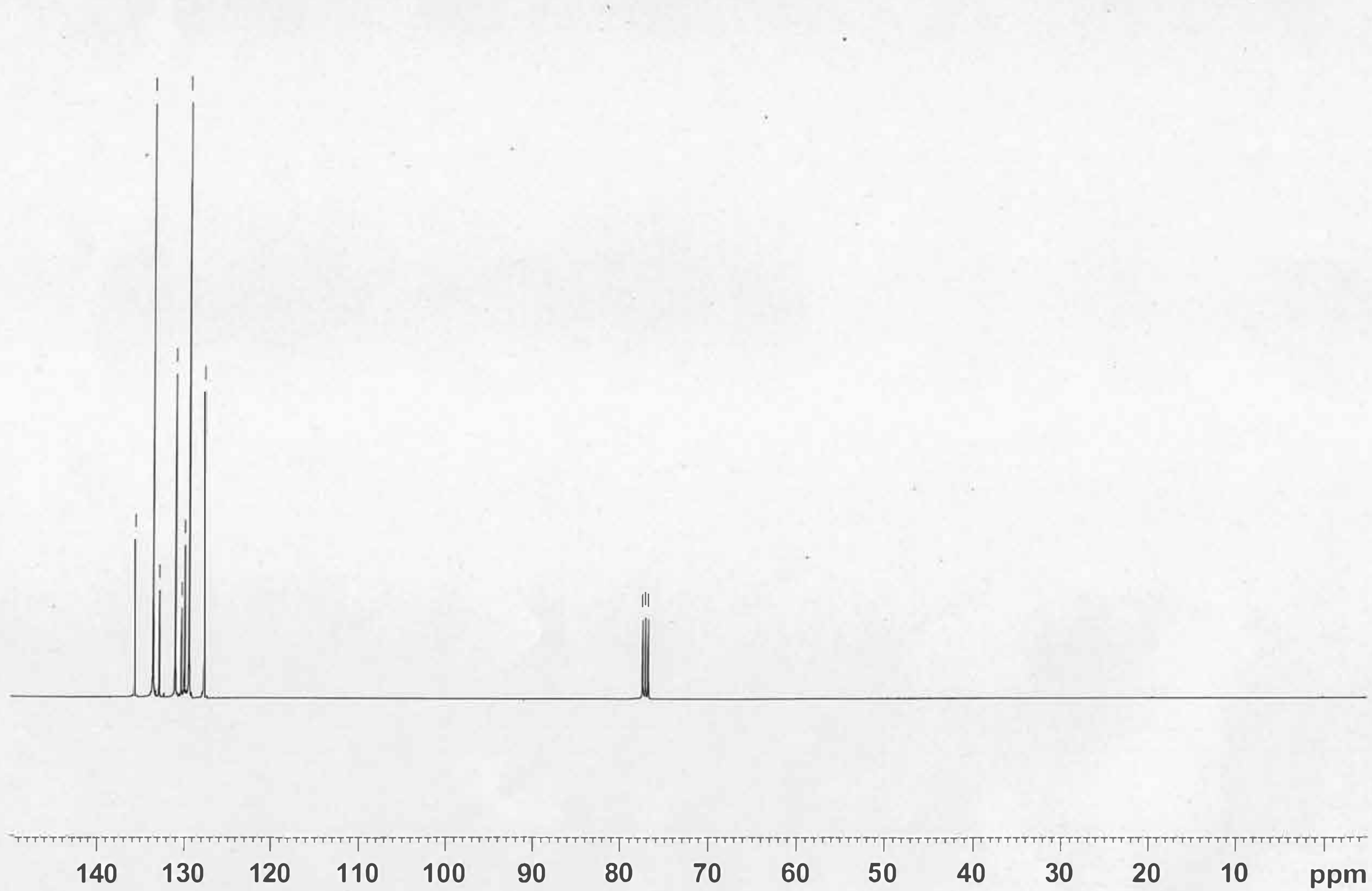

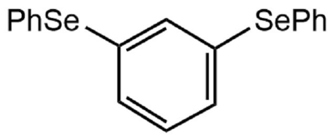

3a

NAME	A23MS222TT	
EXPNO	24061303	
PROCNO	1	
Date_	20240613	
Time	20.18	
INSTRUM	spect	
PROBHD	5 mm CPQNP 1H/	
PULPROG	zgpg30	
TD	65536	
SOLVENT	CDCl3	
NS	2200	
DS	2	
SWH	29761.904	Hz
FIDRES	0.454131	Hz
AQ	1.1010548	sec
RG	129.44	
DW	16.800	usec
DE	18.00	usec
TE	300.0	K
D1	2.00000000	sec
D11	0.03000000	sec
TD0	1	
========	CHANNEL f1 ========	
SFO1	100.6248425	MHz
NUC1	13C	
P1	12.00	usec
SI	32768	
SF	100.6127711	MHz
WDW	EM	
SSB	0	
LB	2.00	Hz
GB	0	
PC	1.40	

13C NMR of Compound 3a

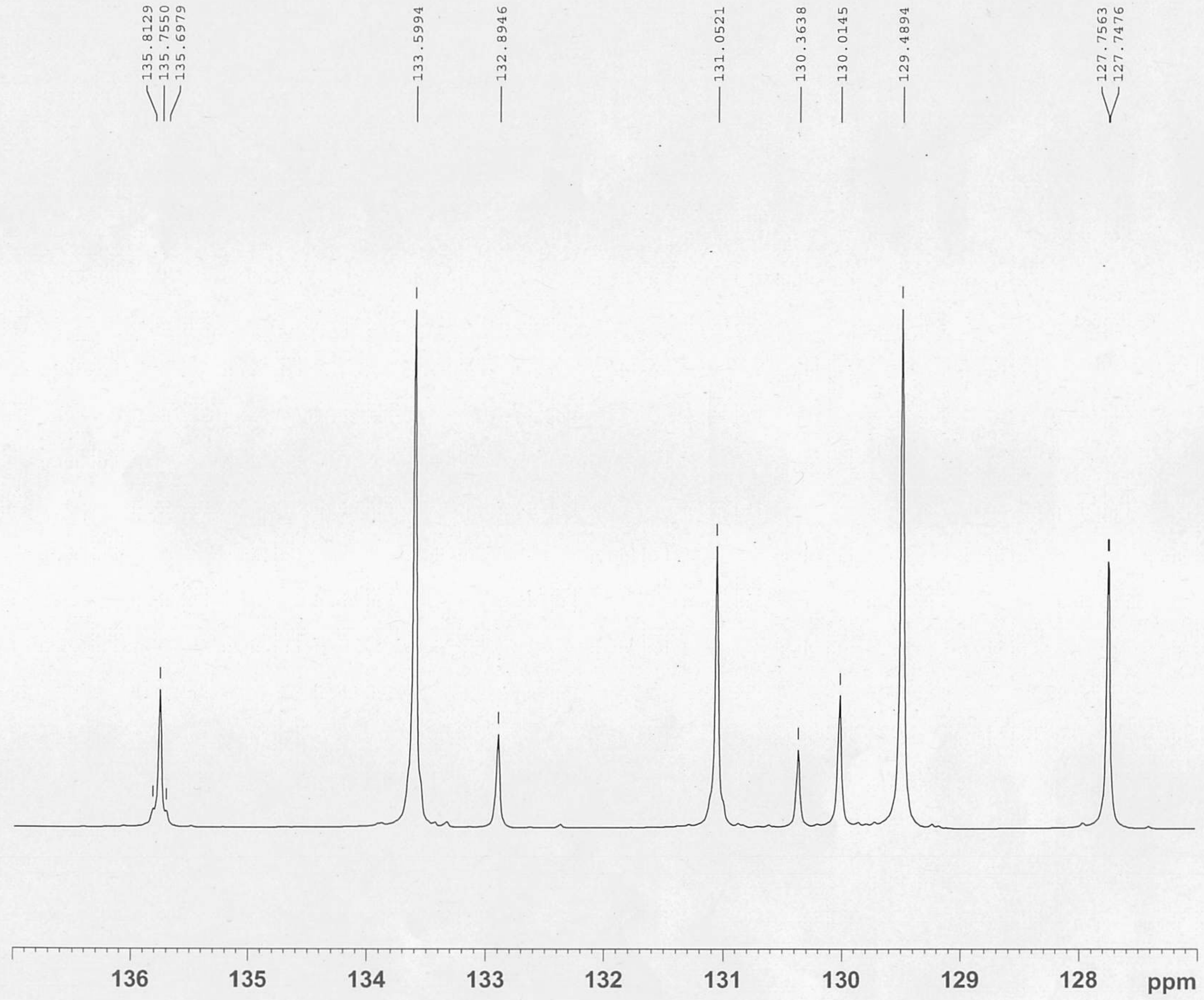

NAME	A23MS222TT	
EXPNO	24061303	
PROCNO	1	
Date_	20240613	
Time	20.18	
INSTRUM	spect	
PROBHD	5 mm CPQNP 1H/	
PULPROG	zgpg30	
TD	65536	
SOLVENT	CDCl3	
NS	2200	
DS	2	
SWH	29761.904	Hz
FIDRES	0.454131	Hz
AQ	1.1010548	sec
RG	129.44	
DW	16.800	usec
DE	18.00	usec
TE	300.0	K
D1	2.00000000	sec
D11	0.03000000	sec
TD0	1	
======== CHANNEL f1 ========		
SFO1	100.6248425	MHz
NUC1	13C	
P1	12.00	usec
SI	32768	
SF	100.6127711	MHz
WDW	EM	
SSB	0	
LB	2.00	Hz
GB	0	
PC	1.40	

77Se NMR of Compound 3a

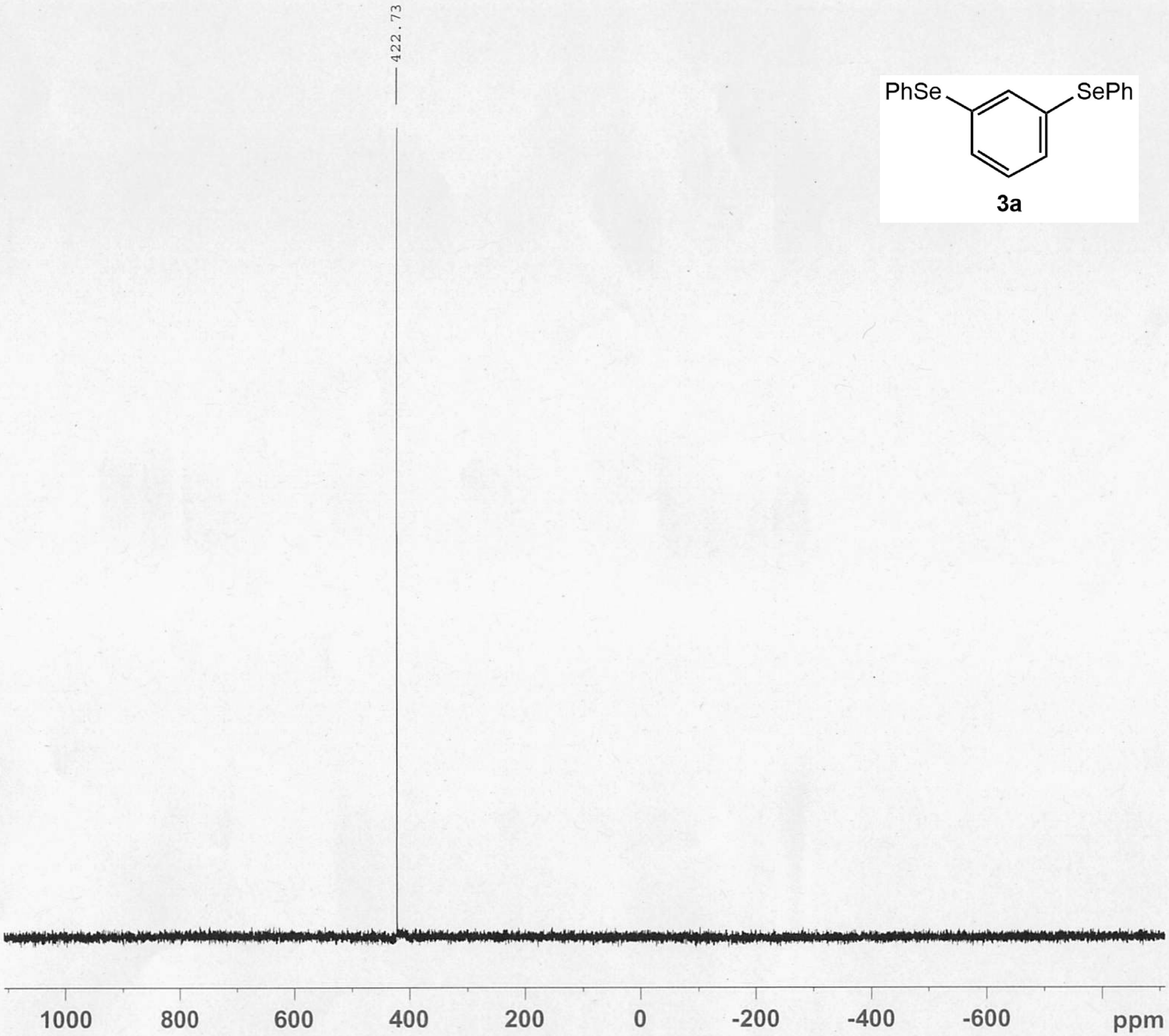

3a

Current Data Parameters
NAME A24MS208ST
EXPNO 24042402
PROCNO 1

F2 - Acquisition Parameters
Date_ 20240424
Time 18.13
INSTRUM spect
PROBHD 5 mm PABBO BB-
PULPROG zgpg30
TD 240000
SOLVENT CDCl3
NS 124
DS 4
SWH 192307.688 Hz
FIDRES 0.801282 Hz
AQ 0.6240000 sec
RG 196.87
DW 2.600 usec
DE 6.50 usec
TE 301.3 K
D1 2.00000000 sec
D11 0.03000000 sec
TD0 1

======== CHANNEL f1 ========
SFO1 95.3728223 MHz
NUC1 77Se
P1 15.00 usec
PLW1 65.00000000 W

======== CHANNEL f2 ========
SFO2 500.0316815 MHz
NUC2 1H
CPDPRG[2 waltz16
PCPD2 80.00 usec
PLW2 16.00000000 W
PLW12 0.36000001 W
PLW13 0.18108000 W

F2 - Processing parameters
SI 16384
SF 95.3633611 MHz
WDW EM
SSB 0
LB 1.00 Hz
GB 0
PC 1.40
